# Resistance training does not increase myocellular garbage dumps: A pilot study on lipofuscin in skeletal muscle fibers of resistance trained young men

**DOI:** 10.14814/phy2.15922

**Published:** 2024-01-31

**Authors:** Daniel Jacko, Lukas Masur, Kirill Schaaf, Jonas Zacher, Käthe Bersiner, Markus de Marées, Wilhelm Bloch, Sebastian Gehlert

**Affiliations:** ^1^ Department of Molecular and Cellular Sports Medicine Institute of Cardiovascular Research and Sports Medicine, German Sport University Cologne Germany; ^2^ Department of Preventative and Rehabilitative Sports and Performance Medicine Institute of Cardiovascular Research and Sports Medicine, German Sport University Cologne Germany; ^3^ Institute of Sport Science, Department for Biosciences of Sports University of Hildesheim Hildesheim Germany; ^4^ Institute of Sports medicine and Sports Nutrition Ruhr University Bochum Bochum Germany

**Keywords:** exercise, fiber types, human skeletal muscle, lipofuscin, SOD

## Abstract

Lipofuscin (LF) is an intracellular aggregate associated with proteostatic impairments, especially prevalent in nondividing skeletal muscle fibers. Reactive oxygen species (ROS) drive LF‐formation. Resistance training (RT) improves muscle performance but also increases ROS production, potentially promoting LF‐formation. Thus, we aimed to investigate if RT of a mesocycle duration increases LF‐formation in type‐I and II muscle fibers and whether RT increases the antioxidant capacity (AOC) in terms of SOD1 and SOD2 content. An intervention group (IG) performed 14 eccentrically accented RT‐sessions within 7 weeks. Vastus lateralis muscle biopsies were collected before and after the intervention from IG as well as from a control group (CG) which refrained from RT for the same duration. LF was predominantly found near nuclei, followed by membrane‐near and a minor amount in the fiber core, with corresponding spot sizes. Overall, LF‐content was higher in type‐I than type‐II fibers (*p* < 0.05). There was no increase in LF‐content in type‐I or IIA fibers, neither for the IG following RT nor for the CG. The same is valid for SOD1/2. We conclude that, in healthy subjects, RT can be safely performed, without adverse effects on increased LF‐formation.

## 1 INTRODUCTION

Although cellular recycling mechanisms are highly effective, incomplete degradation in certain cases can lead to the formation and accumulation of nondegradable molecules (López‐Otín et al., 2013). The most prominent product of such incomplete or impaired recycling mechanisms is Lipofuscin (LF) (Brunk & Terman, 2002; Moreno‐García et al., 2018), which is composed of highly oxidized, covalently bound proteins (30%–70%), lipids (20%–50%), metal ions and sugar residues (Double et al., 2008; Jolly et al., 1995; Moreno‐García et al., 2018). This complex structure renders LF highly cross‐linked, making it nondegradable and leading to its gradual accumulation (Feige & Rudnicki, 2020; Höhn & Grune, 2013). Skeletal muscle fibers are particularly susceptible to LF‐accumulation since they do not undergo further ordinary division. Hence, LF does not become diluted but accumulates increasingly over time (López‐Otín et al., 2013).

Under normal physiological conditions, LF‐accumulation is generally considered unproblematic (Family et al., 2011). Rather, it is associated with the aging process, as demonstrated in various tissues and species (Devi, 2003; Hütter et al., 2007; Koneff, 1886; Nakae et al., 2003; Örlander et al., 1978; Schmucker & Sachs, 2002; Strehler et al., 1959; Tohma et al., 2011). In certain circumstances, especially under pathophysiological conditions (Feeney et al., 2014; Neel et al., 2015, Cataldo et al., 1994; Lv et al., 2011; Moreno‐García et al., 2018) like chronic obstructive pulmonary disease (Allaire et al., 2002), mitochondrial‐ and other myopathies (Lu et al., 2020; Nakae et al., 2003; Terrill et al., 2013; Tohma et al., 2011) or increased reactive oxygen species (ROS) production and cellular redox imbalance in general, LF‐accumulation beyond the norm can be observed (Brunk & Terman, 2002; Höhn & Grune, 2013; Moreno‐García et al., 2018). This, in turn, may disrupt cellular processes and lead to functional impairment. Crucially to note is that, redox imbalance is not solely a result of pathophysiology but is also a hallmark of physical activity like endurance and resistance exercise (Fisher‐Wellman & Bloomer, 2009; Hoppeler et al., 2003; Martinelli et al., 1990). This can be attributed to the fact, that ROS are generated, particularly in energy‐demanding processes by mitochondria or during anaerobic metabolism while converting lactate to pyruvate (Hashimoto et al., 2007; Nikooie et al., 2021). Moreover, mechanical stress, especially due to eccentric resistance exercise is able to induce ROS‐production since it causes diffusion of calcium ions in the sarcoplasm via stretch‐activated channels (Kanzaki et al., 2022). Calcium ions are known to stimulate ROS‐formation in mitochondria and xanthine oxidases (Kanzaki et al., 2022). Then, an imbalance in cellular redox‐state can lead to LF‐accumulation (Brunk & Terman, 2002; Höhn et al., 2011; Höhn & Grune, 2013).

The negative impact of LF becomes evident considering its interference with cellular recycling processes. LF possesses a considerably redox‐active surface, facilitating its binding to subcellular components of the degradation machinery, such as proteolytic enzymes, proteasomes, and lysosomal proteases (Höhn et al., 2011; Höhn & Grune, 2013). This competitive binding may promote LF‐formation or create a vicious circle, where redox imbalance promotes LF‐formation, which, in turn, disrupts proteasomal machineries, leading to further LF‐accumulation (Höhn & Grune, 2013; Yin, 1996). Thus, beyond being seemingly a physiological accompaniment of aging, LF is actively involved in the potential progression of cellular pathological processes, underscoring the significance of examining LF from various perspectives, including the impact of physical exercise on LF‐accumulation in human skeletal muscle.

Skeletal muscle is a highly metabolically active tissue, and increased physical activity can boost its energy turnover rate up to 100‐fold (Hargreaves & Spriet, 2020), potentially promoting LF‐formation. Coupled with its inability to dilute LF‐concentration through mitosis, the significance of potential LF‐formation in skeletal muscle tissue becomes evident (Bouviere et al., 2021; König et al., 2017). Additionally, skeletal muscle is composed of distinct fiber types (I, IIA, and IIX), each differing in metabolic characteristics due to varying mitochondrial content, and thus, susceptibility to LF‐accumulation (Allaire et al., 2002; Örlander et al., 1978). Few studies have specifically investigated LF‐content in different fiber types, particularly in humans (Allaire et al., 2002; Hütter et al., 2007; Örlander et al., 1978) but overall they have shown higher content in type‐I than in type‐II fibers (Allaire et al., 2002; Örlander et al., 1978).

In recent years, resistance training (RT) has gained significance, with numerous reports highlighting its positive effects on human health (Bull et al., 2020; Westcott, 2012). This includes improved physical performance (Henwood & Taaffe, 2005), increased muscle mass (Westcott et al., 2009), positive effects on the cardiovascular system (Strasser & Schobersberger, 2011), and improved insulin sensitivity (Hurley & Roth, 2000). As a result, meantime RT is part of therapy concepts for conditions such as COPD, Parkinson's disease, and myopathies, including Duchenne muscular dystrophy (Brienesse & Emerson, 2013; O'Shea et al., 2009; Phillips & Mastaglia, 2000), where it is considered an effective countermeasure against sarcopenia and strength loss (Brienesse & Emerson, 2013; O'Shea et al., 2009; Phillips & Mastaglia, 2000). Concurrently, RT enhances energy metabolism, particularly anaerobic glycolysis (Goreham et al., 1999), leading to high intramyocellular (Macdougall et al., 1999) and systemic (Wirtz et al., 2014) lactate concentrations. Lactate, or its conversion to pyruvate, has been shown to increase ROS formation (Nikooie et al., 2021), which, as described earlier, can disrupt cellular recycling mechanisms and potentially promote LF‐formation (Bouviere et al., 2021; Höhn & Grune, 2013; Steinbacher & Eckl, 2015).

However, previous studies on LF‐accumulation in human skeletal muscle, especially in the context of exercise or resistance exercise, have been limited. Most of the research was based on rodent experiments and endurance‐oriented exercise. For instance, Basson et al. (1982) examined the influence of lifelong endurance exercise, among other factors, on skeletal muscle and spleen in rats and found significantly higher LF‐content in the trained cohort compared to the sedentary group. This is supported by Faist et al. (2001), who reported higher LF‐content in mice after 6 weeks of endurance exercise. Salminen et al. (1982) examined young mice, running one time for 8 h with two 15 min pauses. They determined a slight but significant increase of LF in skeletal muscle after the exercise (Salminen et al., 1982). There are, however, conflicting findings, as studies by Matthews et al. (2001), Nilsson et al. (2015), and Ravi Kiran et al. (2004) reported a decrease in LF‐content in skeletal or cardiac muscle of animals after endurance training.

To our knowledge, there is no study examining LF in the context of acute resistance exercise (RE) or RT. Only Fridén et al. (1984) analyzed LF, in relation to physical exercise with an emphasis on mechanical stress in human skeletal muscle of young healthy adults. They observed noticeable LF‐appearance 3 days after an acute 30‐min bout of eccentrically oriented exercise on a bicycle ergometer (Fridén et al., 1984). Thus, LF‐accumulation may not only be of significance in pathological situations, but also in healthy populations. Therefore, we assumed that a mesocycle of RT lasting several weeks may lead to increased LF‐accumulation.

Collectively, it is plausible that RT, despite its well‐established health benefits, may also have an adverse impact on muscle fibers, which has largely gone unnoticed thus far, by promoting an increased formation of LF or “muscle aging.”

Considering the limited knowledge regarding potential LF‐accumulation in pure RT models, we analyzed the effect of systematic RT over a mesocycle of 7 weeks on LF‐accumulation in skeletal muscle fibers of healthy subjects and compared it to an inactive control group. We quantified the difference in LF‐content between type‐I and IIA (IIX) fibers and determined the subcellular distribution of LF‐aggregates. Additionally, we examined if RT led to an improvement in antioxidant capacity, which might be an influencing factor for LF‐formation.

## 2 METHODS

### 2.1 Subjects

Twelve male subjects were assigned to either the IG (*n* = 6; 24.2 years ± 4.5; 184.8 cm ± 2.5; 80.3 kg ± 6.8) or the CG (*n* = 6; 24.7 years ± 4.3; 186.7 cm ± 3.3; 82.7 kg ± 4.8). All participants had experience with resistance training, but were not specifically trained in any discipline, endurance, or RT. Subjects were informed about the goal of the study orally as well as in written and signed a corresponding declaration of consent. The study was approved by the ethics committee of the German Sports University Cologne (application #005/2018).

### 2.2 Training intervention

The training, which was only executed by the IG, lasted 7 weeks or a mesocycle and comprised 14 training sessions. One week prior to the start of the training program, the individual training weights were determined. At the beginning of each training session, subjects executed a general warm‐up followed by a specific one (bicycle ergometer: 5 min at 1 W/kg body weight; leg extensions: 10 repetitions with 70% of the 10‐repetition maximum [RM]).

A training session consisted of (1) leg extension (Gym80, Gelsenkirchen, Germany): one set each within the individual 4‐, 8‐, and 16‐RM, (2) leg press (MANG Sportgeräte, Bellenberg, Germany): one set each within the individual 4‐ and 8‐RM, (3) One set of 10 drop jumps (60 cm hight), and (4) a downstair walk: 182 stairs with a height of 18 cm each, whereby two steps were taken at once. The first and the last exercise session (#1 and #14) of the training period consisted of double the volume than the rest, that is, two sets respectively, instead of one. Moreover, a muscle biopsy was taken 1 h after these two sessions. However, these were not considered for the present study.

Resting phase between sets was 120 s and 180 s between exercises. To emphasize on eccentric muscle contractions which is associated with muscular damage and an increased oxidative stress (Kanzaki et al., 2022), which in turn potentially induced LF‐accumulation (Höhn & Grune, 2013; Terman et al., 2010), in leg extension and leg press the movement pattern was conducted with a standardized cadence of 1 s in the concentric‐ and 2 s in the eccentric phase.

In the IG, all subjects trained with a constant load during the training period. The CG was not engaged in RT before the begin of their study period and refrained from RT for a corresponding period of 6 weeks between pre‐ and post‐biopsies (period was 1 week shorter than for IG).

### 2.3 Muscle tissue collection

Muscle biopsies from vastus lateralis were conducted with the percutaneous needle biopsy technique (Bergstrom, 1975). After extraction, the muscle tissue was freed from blood as well as from nonmuscle tissue. The specimen used for Western blotting were directly snap frozen in liquid nitrogen and specimen for immunohistochemistry were snap frozen in isopentane, which was precooled in liquid nitrogen. All samples were then stored at −80°C until further processing.

Biopsies were conducted between 08:00 am and 09:00 am, after an overnight fast. Both, pre‐ and post‐biopsies were taken from the same leg; 5 days before the first and after the last training session, respectively. In order to standardize nutritional intake, a drink containing 20 g proteins, 25.8 g carbohydrates, 13.4 g fat, and 1260 kJ (Fresubin energy drink; Fresenius Cabi, Germany) was ingested 2 h before tissue collection. Drinking water was allowed ad libitum.

### 2.4 Protein extraction

The muscle tissue was lysed in a first step in a 1× Triton X‐100 lysis buffer (pH 7.5) (×10, #9803, Cell Signaling Technology) containing Halt™ Protease and Phosphatase Inhibitor Cocktail (×100, #78440, Thermo Fischer Scientific™) at a 1:100 (vol/vol) ratio and Phenylmethylsulfonylfluorid (PMSF). 2.8 mm and 1.4 mm zirconium oxide beads (CKMix—2 mL, P000918‐LYSKO‐A.0, Bertin Instruments) were added and tubes were placed in a Precellys® 24 tissue homogenizer (Bertin Instruments, Montigny, France). Homogenization was performed with 4 cycles, each at 5800 rpm for 15 s and 120 s of pause in between during which the samples were placed on ice. Then, centrifugation was conducted for 15 min at 15800 rpm and 4°C, resulting in a Triton‐X100 soluble supernatant and an insoluble pellet. The supernatant was transferred to a new microcentrifuge tube. Pellet lysis was done with an urea buffer (4 M urea, 10 mM tris–HCl pH 7.5; 5% SDS; 0.5 mM DTT; 10% glycerol, added with abovementioned protease and phosphatase inhibitors). The pellet was dissolved using three homogenization cycles of 10s, 4500 rpm, and 2 min inter‐cycle pause at room temperature. Finally, both fractions were united to a whole cell lysate. Before, this two‐step lysis method to obtain a whole cell lysate was validated in our lab and showed no difference in comparison to a one step whole cell lysis. Protein concentration was determined and 4x laemmli sample buffer (#1610747, Bio‐Rad), containing 10% beta‐mercaptoethanol was added and samples were heated for 5 min at 95°C.

### 2.5 Western blotting

#### 2.5.1 Antibodies used for Western blotting

Primary: SOD‐1 (FL‐154, # L2105, rabbit polyclonal IgG, 1:500, Santa Cruz Biotechnology), SOD‐2 (FL‐222, # J1405, rabbit polyclonal IgG, 1:500, Santa Cruz Biotechnology). Secondary: anti‐rabbit IgG, HRP‐linked (# 7074S; 1:7500; Cell Signaling Technology).

Equal amounts of proteins (12 μg) for each subject and time point were loaded on a CriterionTM 26‐well, 4%–12% BIS‐TRIS Gel (#3450125, Bio‐Rad) and run with an XT‐MOPS‐buffer (#1610788, Bio‐Rad) with a constant current (Power Pac Basic, Bio‐Rad). Subsequently, the transfer of the gel to a polyvinylidene difluoride membrane (GE Healthcare Life Science, Amersham, UK) were accomplished by semidry blotting (Trans Blot Turbo, Bio‐Rad). The transfer was checked by staining the PVDF membrane with Ponceau S. Membranes were then blocked in 5% nonfat dry milk, dissolved in tris‐buffered saline supplemented with 0.1% Tween20 (TBS‐T), for 1 hour at room temperature. After washing, the membranes were incubated with primary antibodies overnight at 4°C. Then, membranes were washed and incubated with secondary antibodies diluted in TBS‐T containing 5% nonfat dry milk for 1 hour at room temperature. Then, after washing, membranes were incubated for 3 min with an enhanced chemiluminescence assay (ECL‐Kit, GE Healthcare Life Science) and automatically captured (ChemiDoc MP, Bio‐Rad). ImageJ software (v. 1.53j; National Institute of Health) was utilized to assess band densities semi‐quantitatively.

### 2.6 Immunohistochemistry

#### 2.6.1 Antibodies and staining chemicals used for immunohistochemistry

Primary: A4.951 (mouse monoclonal IgG, 1:50, Developmental Studies Hybridoma Bank), 6H1 (mouse monoclonal IgM, 1:50, Developmental Studies Hybridoma Bank), Wheat Germ, DAPI (4′,6‐Diamidino‐2‐phenylindole), DRAQ5 (1,5‐bis{[2‐(di‐methylamino) ethyl]amino}‐4, 8‐dihydroxyanthracene‐9,10‐dione).

Secondary: goat‐anti mouse polyclonal, (H + L) highly cross‐absorbed secondary antibodies (Life Technologies, 555; # A21424 and 633; #A21044: 1:500 in 0.05 mM tris‐buffered‐saline).

LF‐detection and fiber type assignment were performed on different, consecutively cut sections. Sections for LF‐detection were stained for membranes (Wheat Germ) and nuclei (DAPI/DRAQ5) and LF itself was assessed via its autofluorescence which results from the formation of Schiff bases that emerge in the process of LF‐formation (Brunk & Terman, 2002). Consecutive sections were stained for fiber type‐I (A4.951), IIX (6H1; IIA remained unstained), and membranes (Wheat Germ).

The frozen muscle samples were cut in 7 μm thick cross‐sectional slices with a Leica CM 350 S Cryo‐Microtome (Leica Microsystems) and placed on polysine microscope slides (VWR International).

After air drying for 1 hour at room temperature, all slides were placed in −20°C acetone for 8 min, and air died again for 10 min. Then, the samples were blocked for 1 h with 5% bovine serum albumin (BSA; fraction V) dissolved in TBS. Ensuing, samples were incubated with the primary antibody (A4.951) diluted in 0.8% BSA overnight. After washing, samples were incubated with the secondary antibody for 1 h at room temperature and subsequently washed two times for 15 min with TBS. Then, the procedure was repeated from the blocking step for 6H1 (IIX fibers) antibody. Subsequently, for membrane and nuclei staining, samples were incubated for 15 or 6 min at room temperature with Wheat Germ or DAPI/DRAQ5 and washed two times for 15 min with TBS. Finally, the slides were embedded in Aqua‐Poly/Mount and covered with a coverslip.

### 2.7 Quantification of lipofuscin

For LF‐detection, images were taken with a Laser Scanning Microscope (Zeiss LSM 510; Meta confocal microscope; Carl Zeiss) at 40‐fold magnification (Plan‐Apochromat objective 40x/1.0 lens; Carl Zeiss). To identify the corresponding fiber types, images of the consecutive cross sections were taken at 20‐fold magnification (Plan‐Apochromat objective 20×/1.0 lens; Carl Zeiss).

According to Tohma et al. (2011), previous analyses were conducted with manual tracing, which leads to objectivity issues: Subjective assessments of the LF‐area and precision of the tracing influence the analysis of the LF‐aggregates. Thus, they developed an automated method based on a procedure in Fiji is just ImageJ (FIJI) (Schindelin et al., 2012). The evaluation in the present study was also carried out based on this. In order to overcome the problem of different background lightings between the images a specific threshold level was determined and applied for each image of the LF‐sections. The following equation underlie this approach: mean + (n) × standard deviation, where (*n*) represents a specific multiplier (Tohma et al., 2011). In accordance with Tohma et al. (2011), we used the multiplier six. After adjusting the binarized autofluorescence image by the appropriate threshold from this equation, the area of each aggregate were determined by an automated procedure on every captured image of all the muscle sections using ImageJ free software (Schindelin et al., 2012). In the process, granule circularity was set to 0.00–1.00. Cross‐sectional areas of muscle fibers were determined by scanning binary images of cell membranes with the semiautomatic wand tool of FIJI. Consistent with the investigation of Nakae and Stoward (2016), the criterion for exclusion for LF‐assessment was a size under 1.5μm^2^. Furthermore, LF‐aggregates bigger than the subjects average LF‐size plus the threefold standard deviation were excluded.

In order to counter the heterogeneity of the LF‐distribution in the muscle tissue at least 24 images per subject and time point were taken, which ensures an appropriate precision of measurement (Tohma et al., 2011).

### 2.8 Statistics

Statistical analyses were performed using nonparametric methods for datasets based on six subjects. The Wilcoxon matched‐pairs signed rank test was employed for comparing two populations, while the Mann–Whitney test was used for unpaired samples' rank comparison.

For datasets based on 12 subjects, parametric analyses were conducted after testing for normal distribution with the Kolmogorov–Smirnov test. When comparing more than two matched populations, ANOVA with the mixed effects model was applied, accounting for missing values. Correction for multiple comparisons was performed using the false discovery rate control, specifically the two‐stage step‐up model of Benjamini, Krieger, and Yekutieli. Two‐way ANOVA was utilized for comparisons involving multiple factors, with correction for multiple comparisons performed using the Sidak method. The significance level was set at *p* < 0.05 for all analyses. Statistical computations and graph generation were performed using GraphPad Prism (version 8.0.2).

## 3 RESULTS

**FIGURE 1:**
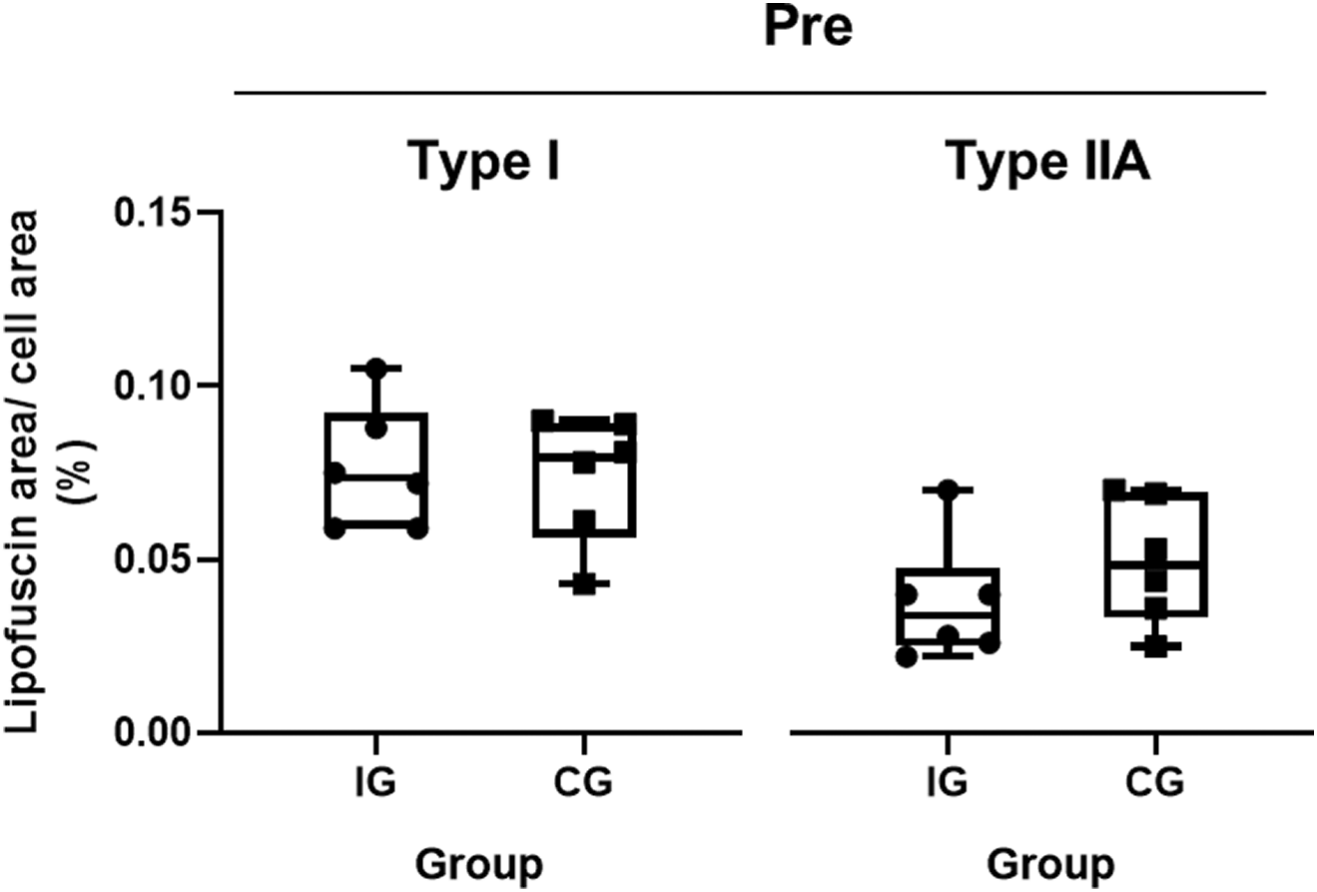
Group comparison of lipofuscin content before the intervention (pre). Comparison of lipofuscin content between the intervention group (IG) and the control group (CG). Box plots represent the minimum to maximum range, the median, and all individual data points.

In a first step, we assessed whether both groups exhibited similar initial conditions (pre) regarding LF‐content in muscle fibers (Figure 1). No significant differences in relative LF‐area were found between the IG and the CG in either type‐I or type‐IIA fibers.

**FIGURE 2:**
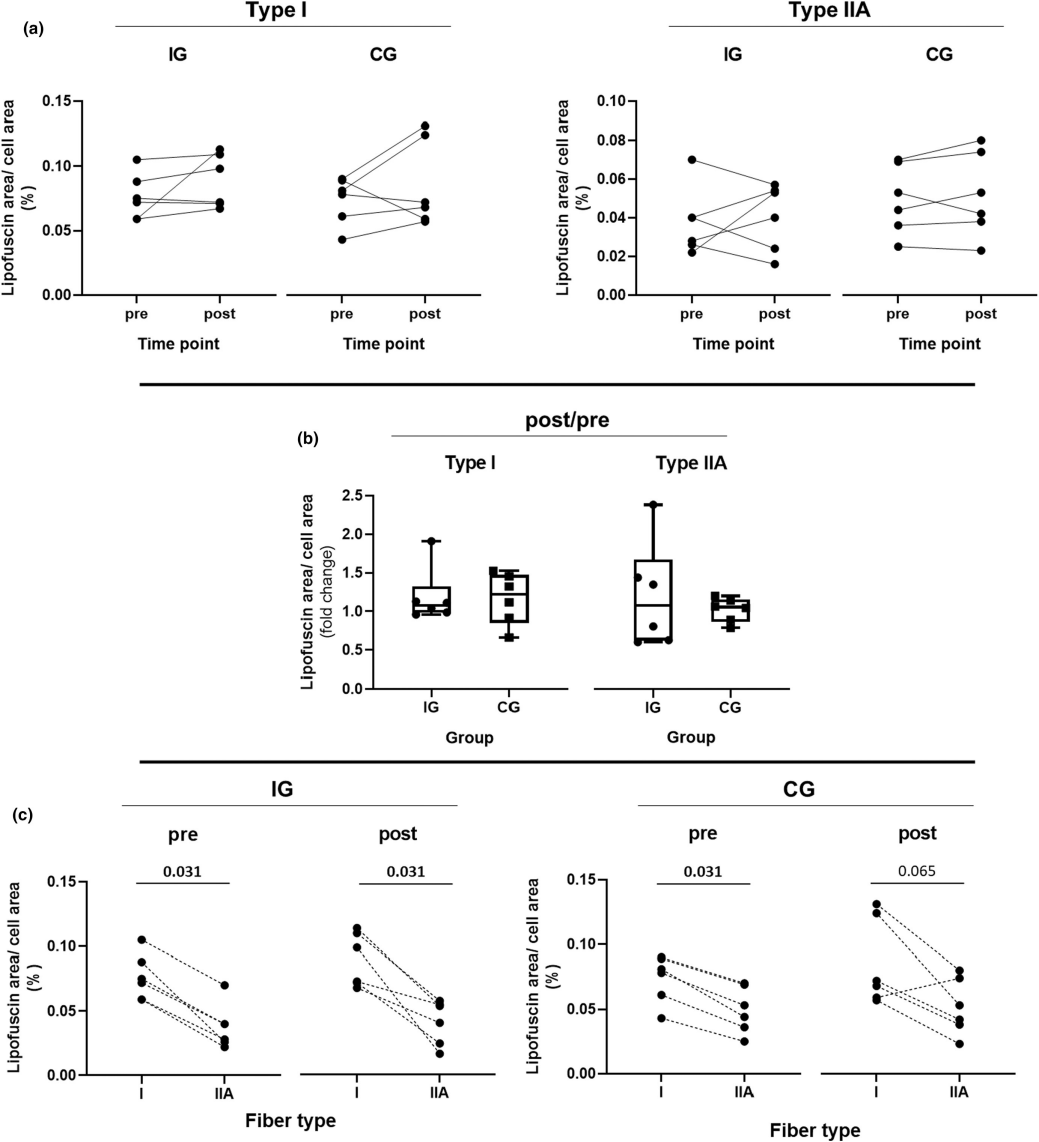
Comparison of lipofuscin content between pre‐ and post‐intervention. (a) Comparison of relative lipofuscin area between pre‐ and post‐training in the intervention group (IG) and control group (CG) separately for type‐I and IIA muscle fibers. (b) Comparison of lipofuscin content change (post/pre) between the intervention group (IG) and control group (CG). Box plots represent the minimum to maximum range, the median, and all individual data points. (**c**) Comparison of relative LF‐area between type‐I and type‐II muscle fibers in IG and CG at pre and post timepoints. The exact *p* values are indicated above the graphs.

Next, we examined whether resistance training resulted in an increase in LF‐content in IG or CG for type‐I and II muscle fibers (Figure 2a). Additionally, we assessed if there were differences in LF‐increase (post/pre) between IG and CG (Figure 2b). There were no significant differences in LF‐area between pre and post, neither for IG nor for CG in both fiber types. Correspondingly, no significant difference in LF‐change was observed between the two groups.

However, the LF‐content was significantly higher in Type‐I fibers compared to type‐II fibers, in both groups (Figure 2c). On average, LF occupied approximately 0.075% of a type‐I fiber and 0.044% of a type‐IIA fiber.

As the LF‐area of a cell results from the sum of its individual LF‐spots, we checked whether there might be a change in the distribution of the LF‐spots. That means: whether the LF‐area before or after loading results from fewer but larger spots or vice versa. Again, no changes were observed between pre and post in IG and CG, nor between the two groups (data not shown).

**FIGURE 3:**
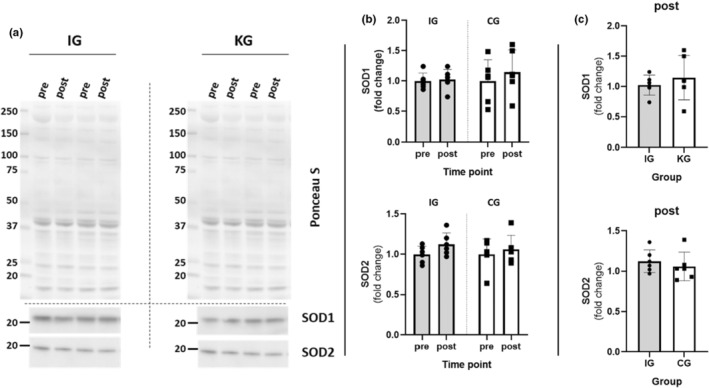
Western blot analysis for copper/zinc superoxide dismutase 1 and 2 (SOD1 and 2). (a) Representative images of Ponceau S stainings (top) and membranes incubated for SOD1 and 2 in Intervention group (IG) and control group (CG). (b) Comparison of SOD1 and 2 between timepoints (pre and Post) in IG and CG. (c) Comparison of SOD1 and 2 content change (post/pre) between IG and CG.

To explore the antioxidant capacity as a possible attenuating factor for LF‐formation (Ismaeel et al., 2019; Sindhi et al., 2013), we examined copper/zinc superoxide dismutase 1 and 2 (SOD1 and SOD2), two well characterized antioxidative enzymes, abating ROS (Figure 3). Once again, no significant differences were found between pre‐ and post‐training in IG and CG (Figure 3b), nor between IG and CG (Figure 3c).

**FIGURE 4:**
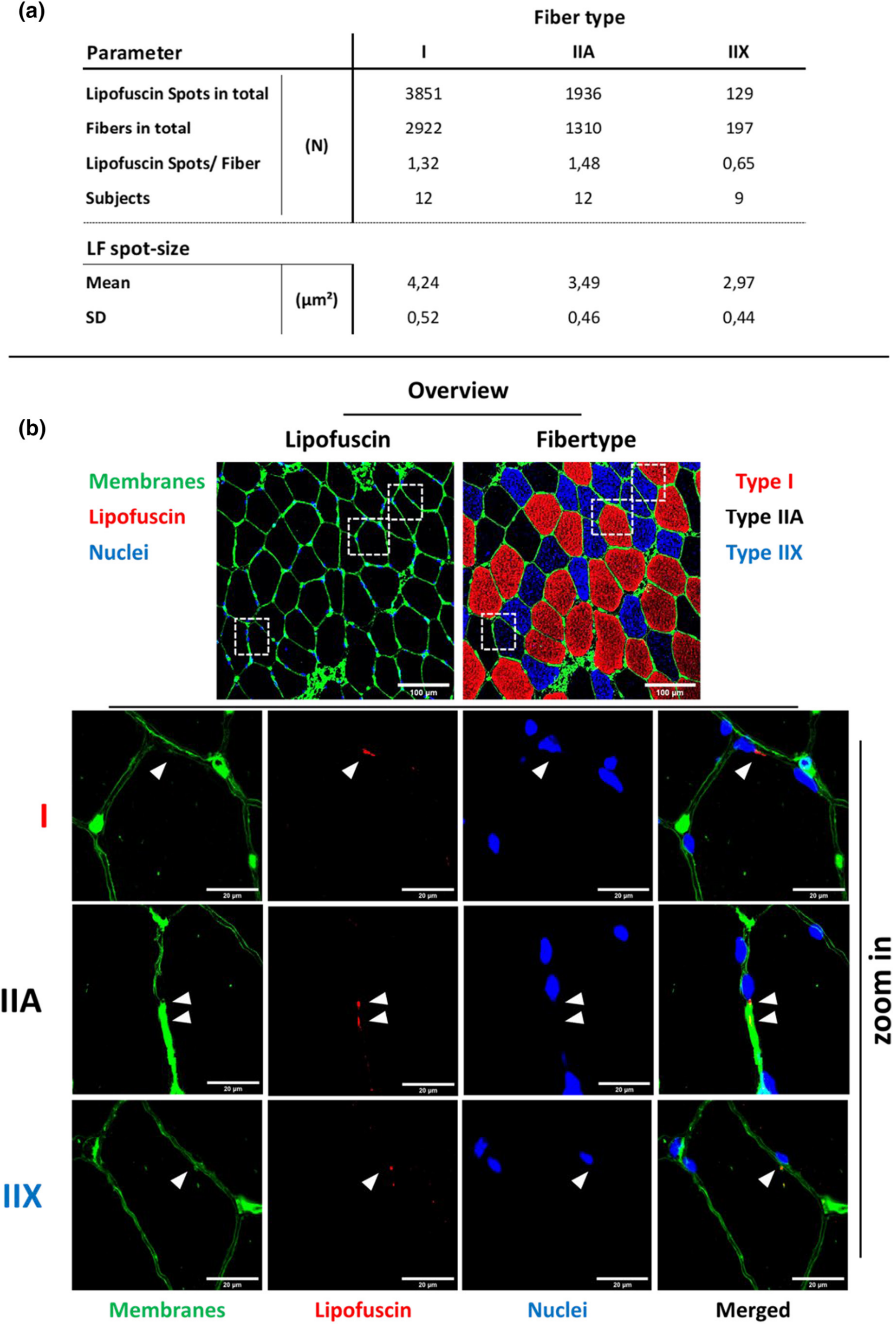
Descriptive statistics. (a) Overview of the total numbers of analyzed Lipofuscin spots and fibers separated in type‐I, IIA, and IIX. The numbers below the dashed line (LF‐spot size) are generated from the mean value from the individual subjects. (b) Representative images of lipofuscin occurrence in type‐I, ‐IIA, and ‐IIX fibers. White arrow heads show lipofuscin localization. Box plots represent min to max, the median and all values.

Since no significant variance was detected in the tested parameters regarding group or timepoint factors, we combined the data sets (IG + CG + pre + post) for further analysis, while separating the data based on fiber type (Figure 4a). This approach now allowed us to include type‐IIX fibers, which were present in nine out of 12 subjects, in statistical analysis.

**FIGURE 5:**
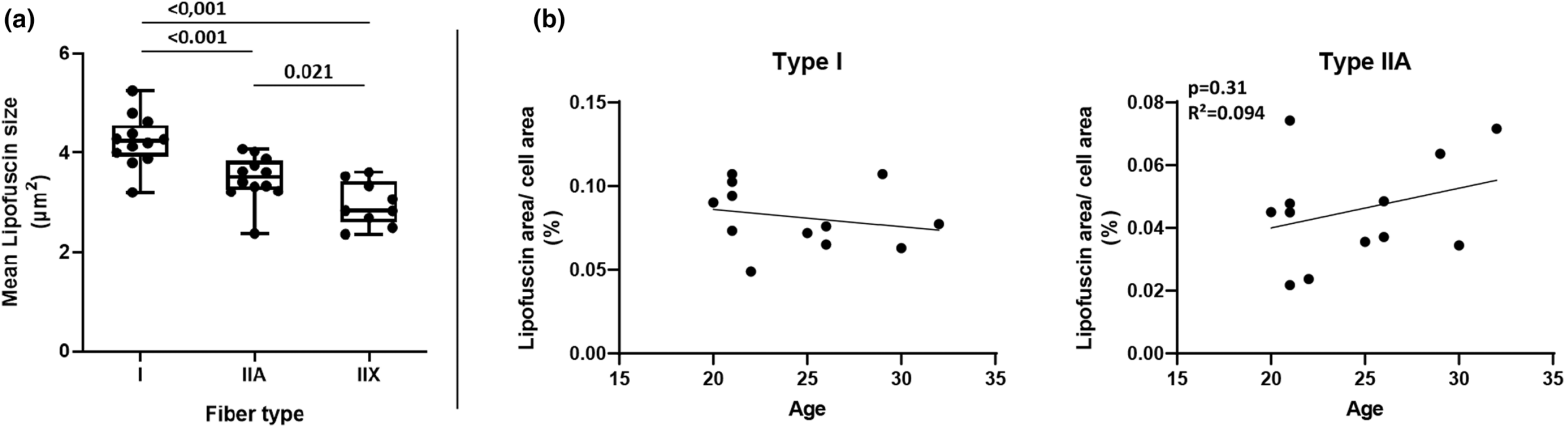
Statistics on combined data sets. Subjects from the intervention group (IG; *n* = 6) and the control group (CG; *n* = 6) were combined, and data from pre‐ and post‐intervention were averaged for each subject. (a) Comparison of absolute lipofuscin size between type‐I, IIA, and IIX fibers. (b) Testing for correlation between age and relative lipofuscin content in type‐I and type‐IIA fibers. Box plots show the minimum to maximum range, the median, and all individual data points. The exact *p* values are indicated above the graphs.

When considering LF‐size, we found differences not only in the sum of LF‐spots (area) relative to fiber area (LF‐Area/Fiber‐Area; Figure 2c), but also in the mean size of individual spots between the three fiber types (I > IIA, I > IIX *p* < 0.001; IIA > IIX, *p* < 0.05; Figure 5a).

As LF is described to accumulate as a consequence of aging in cells which do not undergo ordinary cell division, we also explored whether there was a correlation between the age of subjects and the relative LF‐area related to fiber area in type‐I and ‐IIA fibers. However, no significant correlation was found in subjects ranging in age between 20 and 32 years, neither for type‐I (*p* = 0.48; *R* ^2^ = 0.051) nor for type‐IIA fibers (*p* = 0.31; *R* ^2^ = 0.094). (Figure 5b).

**FIGURE 6:**
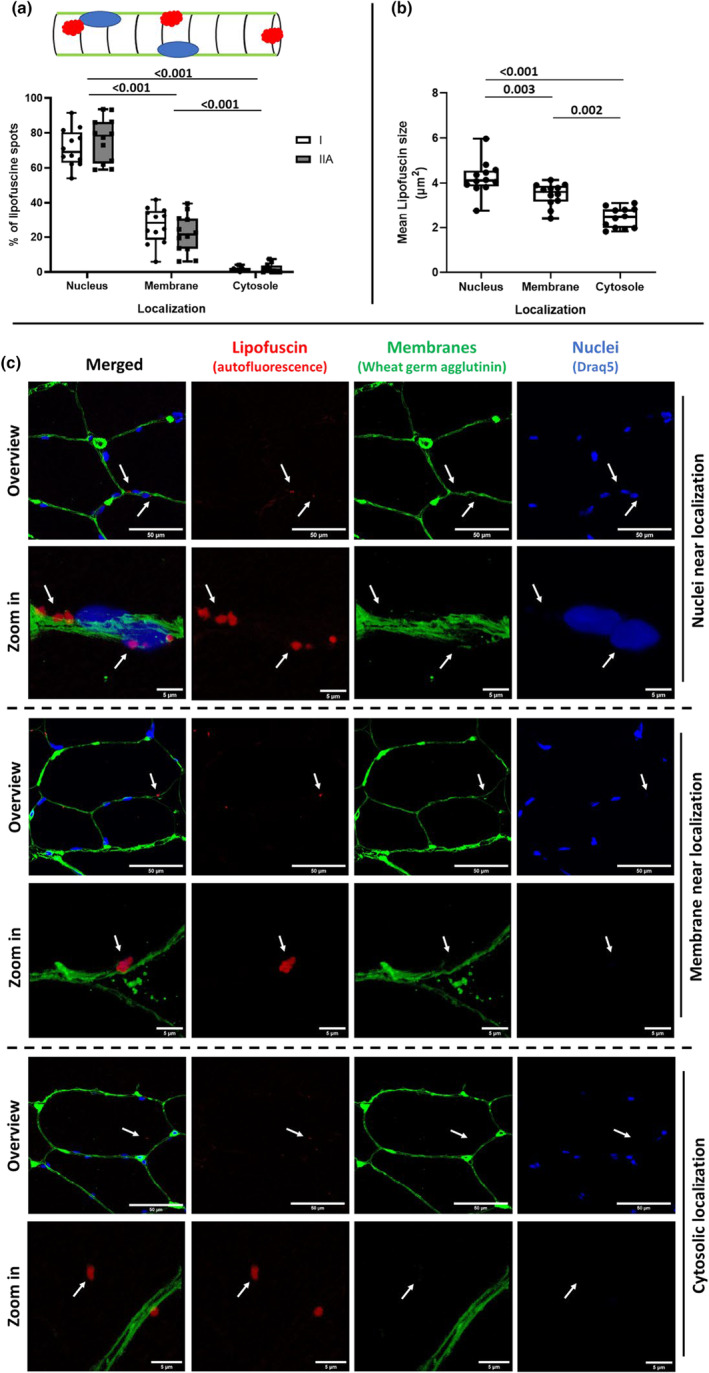
Demonstration of lipofuscin localizations within human skeletal muscle fibers. (a) Quantification of lipofuscin occurrence in type‐I and IIA fibers, including a simplified illustration. (b) Mean lipofuscin area depending on localization. (c) Representative images of lipofuscin showing localization in the vicinity of a nucleus, membrane alone, and a cytosolic localization without any association to membrane or nucleus. The exact *p* values are indicated above the graphs.

Finally, we examined the localization of LF within muscle fibers and observed three main categories. The majority of LF (71.4% in type‐I; 76.1% in IIA) was found in direct vicinity of a nucleus, which is close to the plasma membrane due to peripheral nuclei in skeletal muscle fibers. Additionally, some LF was located near the plasma membrane without a nearby nucleus (26.9% in type‐I; 21.9% in IIA), and only a small fraction appeared freely in the cytosol or in the core of the fibers with no association to the nucleus or membrane (type‐I: 1.7%; type‐IIA: 1.9%). The size of LF‐spots differed based on their localization, with spots in proximity to the nucleus being the largest in terms of area (4.23 ± 0.75 μm^2^) and significantly different from solely membrane‐associated (3.48 ± 0.51 μm^2^; *p* < 0.01) and cytosolic localized spots (2.44 ± 0.46 μm^2^; *p* < 0.001). The latter were the smallest and also differed from purely membrane‐associated ones (*p* < 0.01) (Figure 6).

## 4 DISCUSSION

RT has well‐established beneficial effects on overall health and is also employed to support therapy for various diseases, such as Parkinson's, myopathies, and COPD (Brienesse & Emerson, 2013; O'Shea et al., 2009; Phillips & Mastaglia, 2000; Westcott, 2012). Simultaneously, RT increases the production of ROS, which is a key driver of LF‐accumulation. LF is recognized as a hallmark of cell or tissue aging (Brunk & Terman, 2002; Schmucker & Sachs, 2002; Terman & Brunk, 1998; Yin, 1996). Until now, it has not been elucidated whether RT promotes increased LF‐formation, potentially accelerating the natural aging process of muscle fibers.

Therefore, we investigated if a 7‐week RT mesocycle would augment LF‐content in healthy male subjects. We compared LF‐content in muscle fibers in an IG between pre‐ and post‐RT. Additionally, we compared LF‐content between the IG and a CG that did not undergo RT during a 6‐week period. We are the first showing, that a RT mesocycle does not augment LF‐content in healthy male subjects (Figure 2). Moreover, based on quantitative data from human skeletal muscle, we demonstrate that the extent of LF‐covered area varies between distinct fiber types, with type‐I fibers having 1.7 times higher LF‐content compared to type‐II fibers (Figure 2c). Additionally, we showed, that not only the LF‐area/cell area differs between fiber types, but also the mean single spot size (Figure 4a and Figure 5a). Finally, we determined three main localizations of LF‐aggregates, with the majority found in direct vicinity of nuclei and cell membranes, followed by sole membrane vicinity, and only a negligible number in the fiber core (cytosol), away from the nuclei or sarcolemma (Figure 6). In addition, we determined that the individual LF‐spots differ in size, depending on their localization, which corresponds to the order mentioned above.

LF‐accumulation is a well‐established age‐dependent alteration in post‐mitotic cells and those which do not undergo ordinary cell division, including neurons, myocardial cells, liver cells, and skeletal muscle fibers (Brunk & Terman, 2002; Jolly et al., 1995; Jung et al., 2007; Moreno‐García et al., 2018; Terman et al., 2010; Terman & Brunk, 1998). Previous investigations have shown higher LF‐contents in individuals with Alzheimer's and Parkinson's diseases, COPD, Pompe disease, and other myopathies, compared to age‐matched healthy subjects (Allaire et al., 2002; Moreno‐García et al., 2018; Nakae et al., 2003; Neel et al., 2015; Tohma et al., 2011).

To date, no study has specifically examined LF‐formation in the context of RT. However, Fridén et al. (1984) conducted a study, subjecting participants to one bout of endurance‐type exercise with an augmented eccentric contraction pattern on a modified bicycle ergometer. This exercise mode was designed to induce mechanical stress and muscle damage in contrast to common endurance exercises. Since mechanical stress is a primary attribute of strength training, it can be somewhat comparable to our loading type. Fridén et al. (1984) reported increased LF‐spots in skeletal muscle 3 days after the exercise, but not 1 h or 1 day. They proposed two possible explanations: (1) Acutely increased protein degradation due to noticeable sarcomeric damage and (2) Gradual increase in protein turnover.

We did not detect any LF‐increases, even after multiple training sessions. What could be the possible causes for the contrary findings between their and our study? Despite clear differences in study designs, some similarities exist. Fridén et al. (1984) acknowledged the damage and subsequent protein degradation processes as a key factor of LF‐formation. We observed clear signs of sarcomeric damage, too, which was evident already 1 h after the initial RE‐bout (unpublished data). Currently, it is believed that the autofluorescence associated with LF arises from complex crosslinking of different molecules, rendering it an indigestible aggregate (Feige & Rudnicki, 2020; Moreno‐García et al., 2018). Thus, a single exercise‐bout causing muscle damage should lead to LF‐accumulation, and multiple such stimuli, as in our study, should potentially exacerbate this effect. Unfortunately, it was not in the scope of our investigation to examine LF‐formation acutely after a single bout of unaccustomed exercise. Thus, further investigations are required to clarify occurrence of LF as a result of a single unaccustomed exercise in the context of muscle damage.

ROS, which can disturb autophagy, are considered the main source of LF‐formation in the vast majority of studies (Allaire et al., 2002; Höhn et al., 2011; Höhn & Grune, 2013; Hoppeler et al., 2003; Martinelli et al., 1990; Moreno‐García et al., 2018; Nakae et al., 2003; Neel et al., 2015; Terman et al., 2010; Tohma et al., 2011). Physical exercise and RT increase energy metabolism and induce mechanical stress (Hargreaves & Spriet, 2020; Jones & Rutherford, 1987; Rindom & Vissing, 2016; Wirtz et al., 2014), both of which stimulate ROS formation and may initiate oxidative stress (Bouviere et al., 2021; Fisher‐Wellman & Bloomer, 2009; Höhn & Grune, 2013). On the contrary, RT has been shown to upregulate antioxidant capacity in skeletal muscle (Bailey et al., 2007; Child et al., 1999; Parise et al., 2005). This upregulation ameliorates exercise‐induced increases in ROS production and oxidative stress (Bailey et al., 2007; Fisher‐Wellman & Bloomer, 2009; Ismaeel et al., 2019).

To explore whether upregulation of antioxidant capacity resulting from RT may act as a protective factor against LF‐formation in our study, we tested for potential upregulation of superoxide dismutase 1 and 2 (SOD1 and SOD2). These enzymes are among the most important protective mechanisms against oxidative stress (Sindhi et al., 2013). However, we did not detect any changes in the levels of these enzymes in either group.

This observation is consistent with several other studies that failed to detect oxidative stress after RT (Bloomer et al., 2005; Hellsten et al., 1997; Liu et al., 2005; McAnulty et al., 2005; Ramel et al., 2004). Furthermore, Fisher‐Wellman and Bloomer (2009), Radák et al. (1997), and Witt et al. (1992) stated that the volume, intensity, and type of training are crucial factors for initiating oxidative stress and presumably LF‐formation. Thus, it is plausible to assume that the RT applied in our study did not provoke an oxidative stimulus sufficient to overcome the existing antioxidant protection.

Moreover, the cellular antioxidative system is very versatile and consists of multiple different enzymes, such as glutathione peroxidase, glutathione reductase, catalase, and other antioxidant molecules (Birben et al., 2012; Fisher‐Wellman & Bloomer, 2009). It is possible that alternative antioxidant factors influenced by RT might have affected LF‐formation. Unfortunately, we were not able to directly measure ROS formation itself.

Another consideration for the absence of LF‐accumulation is that RT led to increased enzymatic activity rather than an increased amount of enzymatic protein content. This finding aligns with a study by Mesquita et al. (2021), who observed reduced lipid oxidation and increased content of mRNA coding for antioxidant enzymes but no increase in protein content after RT. They proposed that the antioxidative system might adapt by increasing enzymatic activity rather than protein content after exercise, leading to enhanced antioxidant efficiency and potentially preventing LF‐formation in our case (Mesquita et al., 2021).

Previous studies based on qualitative examination have shown LF‐aggregates in the region of subsarcolemmal nuclei (Jung et al., 2007; Moreno‐García et al., 2018; Nakae et al., 2003). Our evaluation refines those findings, revealing that the localization of LF‐aggregates can be further subdivided (nucleus vicinity, purely subsarcolemmal with no nucleus nearby, centralized/cytosolic). To our knowledge, the present study is the first to demonstrate quantitatively different subcellular localizations of LF in human skeletal muscle fibers and that the mean size of single LF‐spots differs depending on these localizations. The perinuclear location is supposed being attributed to the motility of lysosomes towards nuclei during the process of autophagy (Ballabio & Bonifacino, 2020; Yim & Mizushima, 2020). In the vicinity of nuclei most autophagosome‐lysosome fission occurs and lysosomes are more acidic, which improve enzymatic function (Ballabio & Bonifacino, 2020; Yim & Mizushima, 2020). Furthermore, lysosomes tether and communicate with perinuclear Golgi complex and endoplasmic reticulum (Ballabio & Bonifacino, 2020). The pure‐subsarcolemmal location could be attributed to the subsarcolemmal area's abundance of mitochondria, known to be a crucial source of ROS (Allaire et al., 2002). The central localization of LF appears to be very rare and, to our knowledge, has not been explicitly described, particularly for human skeletal muscle. Unfortunately, there is currently no corresponding data basis for an explanation of this phenomenon.

Early fine‐structure skeletal muscle studies determined an increased accumulation of LF in type‐I muscle fibers compared to type‐II muscle fibers (Örlander et al., 1978; Shimasaki et al., 1977). Moreover, Allaire et al. (2002) examined different LF‐contents in the skeletal muscles of COPD patients compared to controls and noted higher LF‐accumulation in type‐I fibers. This finding aligns with the results of our study. The difference in LF‐accumulation between muscle fiber types is believed to be attributed to the higher oxidative metabolism of type‐I muscle fibers (Allaire et al., 2002; Örlander et al., 1978). Further investigations support this assumption: According to current studies, the mitochondrial respiratory chain, particularly complexes I–III, is a primary source of ROS generation (Díaz‐Vegas et al., 2015). Thus, the higher density of mitochondria and respiratory chain enzymes in type‐I muscle fibers could be a key reason for the differences in LF‐accumulation between fiber types (Allaire et al., 2002; Örlander et al., 1978).

Additionally, we found that the mean size of single LF‐spots differs between the fiber types. Various mean sizes of LF‐spots have been identified in tissues like optic nerve (3–5 μm^2^) (Fernandez De Castro et al., 2013), brain (3–5 μm^2^)^4^, pial artery (0.88–1.97 μm^3^) (Hakvoort et al., 2021), retinal pigment epithelium (0.75 μm^2^) (Biesemeier et al., 2011), hepatic tissue (3.3–10.2 μm^2^) (Tauchi et al., 1980), or muscle tissue (4.31–9.85 μm^2^) (Nakae & Stoward, 2016). The latter investigation by Nakae and Stoward (Nakae & Stoward, 2016) analyzed skeletal muscle fibers of healthy and dystrophic mice by using similar image capture and image analysis methods like our study. The authors suggested that their derived function of LF‐aggregate sizes might be a sensitive measure of chronic oxidative stress (Nakae & Stoward, 2016). Accordingly, as already stated before, the different capacities of oxidative metabolism (Allaire et al., 2002) in distinct fiber types could explain the varied size of individual LF‐aggregates, with type‐I fibers showing the largest aggregates compared to type‐IIA and type‐IIX fibers.

It is known, that LF‐aggregates increase with aging (Family et al., 2011; Schmucker & Sachs, 2002; Sheldrake, 1974). In our study, we failed to find a correlation between LF‐content and the age of our subjects. Strehler et al. (1959) and Frcpa McKelvie et al. (1999) showed in sedentary subjects, that LF only starts to increase significantly at a more advanced age (40–50). Since we examined individuals at 20–32 years of age, our finding is very plausible.

It must be noted that the size information given here for the LF‐aggregates must always be seen in the context of the analysis method. This has a certain influence on the dimensions, as Tohma et al. (2011) have previously shown. Electron microscopy is particularly suitable for exact measurements. This, in turn, has the disadvantage that only small, very selected areas can be taken into account. At the same time, however, we have seen that the size of the units can vary considerably from place to place.

In summary, our study is the first to show that repeated resistance exercise does not lead to increased LF‐formation during a 7‐week RT mesocycle. Moreover, we contribute new data on the subcellular distribution of LF. Our study reveals that most LF in skeletal muscle fibers accumulates in the vicinity of nuclei. However, a subpopulation can also be found directly beneath the sarcolemma without nucleus vicinity and further centralized in the fiber core (cytosolic). Furthermore, our findings demonstrate not only a larger LF‐covered area in type‐I fibers compared to type‐II, but also differences in the mean size of single LF‐spots between fiber types.

In conclusion, RT mesocycles can be applied without concern for adverse effects on LF‐formation, at least in healthy, younger subjects. Nevertheless, further investigations are warranted to examine whether this safety also applies to individuals with pathophysiological problems or redox imbalance and older populations.

## CONFLICT OF INTEREST STATEMENT

None of the authors has any conflict of interest to disclose.

## ETHICS STATEMENT

We confirm that we have read the Journal's position on issues involved in ethical publication and affirm that this report is consistent with those guidelines.
